# First report of Tarantula-parasitic nematode *Tarantobelus jeffdanielsi* from Los Angeles, California

**DOI:** 10.17912/micropub.biology.000825

**Published:** 2023-04-27

**Authors:** Anil Baniya, Julie Ngov, Kyle Anesko, Adler R Dillman

**Affiliations:** 1 Department of Nematology, University of California, Riverside

## Abstract

*Tarantobelus jeffdanielsi* is a recently described nematode parasite of tarantulas, originally isolated from a tarantula breeder in Virginia Beach, VA. We describe a new case of this parasite infecting tarantulas at a breeding facility in Los Angeles, California. Nematodes were isolated from the oral cavity of a captive bred *Psalmopoeus iriminia* commonly referred to as a Venezuelan sun tiger tarantula. rDNA sequencing was conducted to identify the species and generate a phylogeny tree.

**Figure 1. Molecular and morphological identification of T. jeffdanielsi LA:**
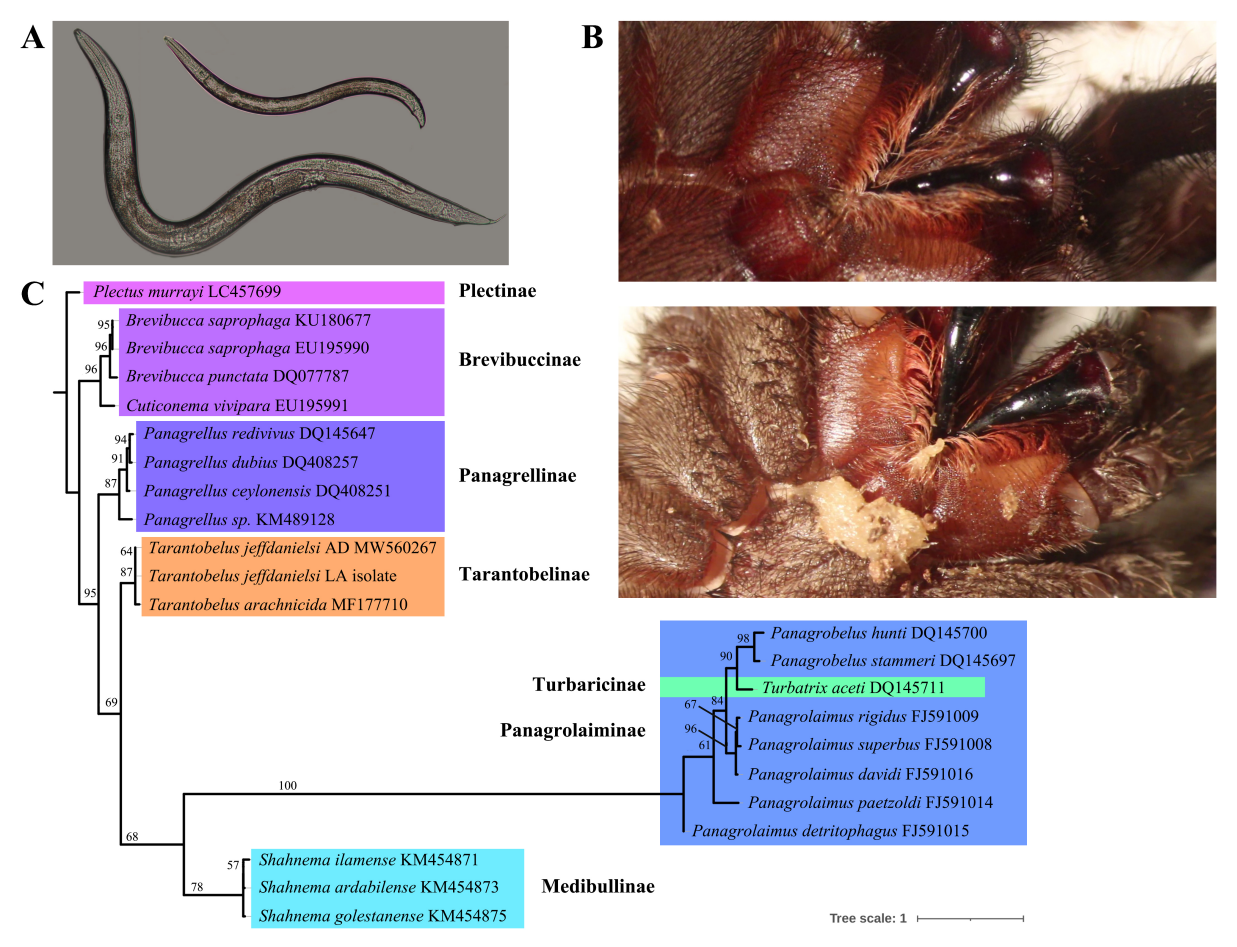
**A.** Morphological characterization of males and females of *T. jeffdanielsi* LA using light microscopy. Females are relatively bigger than males. **B.** The morphology of the mouth part of a healthy (top) and nematode-infected tarantula (bottom). **C.** Maximum likelihood phylogenetic tree inferred based on publicly available nematode sequences and newly sequenced *T. jeffdanielsi* LA isolate targeting the 28S rDNA D2/D3 domain. The phylogenetic tree was run with a bootstrap value of 1,000. The number at the branches represents support values for the nodes. Different subfamilies are grouped and named into different colored boxes.

## Description

**Introduction**

Nematodes are diverse and ubiquitous microscopic eukaryotic organisms that have a significant global impact on ecosystems, humans, agriculture, and animal health (Bogale et al., 2020) . It is estimated that there are more than a million species of nematodes, with only about 0.01% of them being identified and described to date (Blaxter and Koutsovoulos 2015) . Few parasitic nematode species that infect spiders have been described (Poinar 1987) . Wild-caught and captive-bred tarantulas (Theraphosidae) are kept as pets. Pet tarantulas are often described as calm, quiet, low maintenance, visually charismatic, and long-lived invertebrate pets. Because of these features pet tarantulas are in high demand, and an increasing number of people are keeping these exotic animals as pets. There have been reports on oral infection of nematodes affecting valuable collections of tarantulas worldwide. Similarly, there are frequent reports of tarantula infection by nematodes from individual collectors on online hobbyist forums such as Arachnoboards (https://arachnoboards.com). But very few of these infections have been verified or reported in peer-reviewed literature. An initial effort to study and control nematode infections in tarantulas was conducted in 2009, using different combinations and doses of antibiotics, however none of the treatments were effective in controlling nematodes as all the nematode infections lead to the death of infected tarantulas (Pizzi 2009) .

*Tarantobelus arachnicida* was the first nematode species documented to infect tarantulas in 2017 from Poland (Abolafia and Peña-Santiago 2018). In 2019, a case of tarantula infections at a breeding facility in Virginia Beach, VA involved a new species, *Tarantobelus jeffdanielsi* AD isolate (Schurkman et al., 2022) . In November 2022, a tarantula breeder located in Los Angeles (LA), California observed several deaths in their tarantula collection. Diseased tarantulas displayed lethargic behavior and loss of appetite. The diseased tarantulas were received at the Department of Nematology at the University of California, Riverside. We were able to recover the nematodes from the oral cavity of a captive bred *Psalmopoeus iriminia* (common name: Venezuelan sun tiger tarantula). Further diagnosis of the identified nematodes using rDNA sequencing revealed the identity of the nematode as *T. jeffdanieslsi* . In this report, we document a second case of *Tarantobelus jeffdanielsi* infection isolated from a breeding facility in Los Angeles, California.

**Results**

The blast result revealed that the 28S sequence of the new isolate is 99.86% similar to that of *T. jeffdanielsi* AD isolate. Similarly, the ITS sequences of the two isolates were 100% similar to each other. Based on the 28S phylogeny, the new isolate is placed together with *T. jeffdanielsi* AD isolate next to *T. arachnicida* into the Tarantobelinae sub-family clade. The sister clades of the new species are Turbatricinae, Panagrolaiminae, and Medibullinae. A morphological analysis comparing the new isolate to the description of the isolate from Virginia also revealed that the new isolate has similar morphology as that of *T. jeffdanielsi* AD isolate as described by Schurkman et al. (2022). Based on molecular and morphological characteristics we concluded that the new isolate is *T. jeffdanielsi* LA isolate. Sequences generated as part of this study were deposited in GenBank with the following accession numbers: *T. jeffdanielsi* LA (OQ702343) for D2–D3 rDNA sequences, *T. jeffdanielsi* LA (OQ703326) and *T. jeffdanielsi* AD isolate (OQ703327) for ITS sequences.

**Discussion**

Nematodes significantly impact the health and vigor of tarantulas, as evidenced by the symptoms observed in tarantulas infected with nematodes. However, the complete information about the origin of infection, biology, host range, and the mechanism of infection is not known. After the first discovery of this nematode in 2019 in VA beach, Virginia, we again encountered this nematode in LA. The two unrelated cases suggest that these infections may be more widespread than previously speculated. Although we only observed nematodes outside the tarantula's oral cavity, the infected tarantula showed clear symptoms of infection. This leads us to speculate that there might be a bacterial associate of the nematode responsible for the symptoms, which requires further testing and validation. Once a nematode infection has taken hold in a tarantula, no successful treatment has been documented, though topical application of a readily available antihelminthic such as pyrantel pamoate, which is used to treat pinworm, may be helpful. Though additional research is needed. Previous research has shown that *T. jeffdanielsi* successfully infected several different species of tarantulas as well as wax worms ( *Galleria mellonella* ), indicating that this nematode may have a broad host range (Schurkman et al., 2022) . We conclude that *T. jeffdanielsi* is a fascinating parasitic nematode with distinctive biology that warrants further investigation.

## Methods

**Nematode collection**

A diseased sub-adult male tarantula ( *Psalmopoeus iriminia* ) was subject to CO _2_ anesthesia. Nematodes were recovered directly from the oral opening using a number 5 brush on a 1% agar plate. From the initial plate nematodes were transferred to nematode growth media (NGM) plates (3 g NaCl, 2.5 g Peptone, 20 g agar, 10 ml Uracil [2 g/L], 975 ml DI water, autoclave, 25 ml 1 M KPO4 [pH 6.0], 1 ml 1 M MgSO4, 1 ml 1 M CaCl2, and 1 ml cholesterol [5 mg/ml in ethanol]) seeded with *Escherichia coli* OP50; and incubated at 17 ˚C.

**DNA sequencing and phylogenetic analyses**

After 2 weeks of culture in NGM plates approximately 20 nematodes were picked from the NGM plates, and DNA extraction was done using the proteinase K protocol. Briefly, approximately around 20 nematodes were transferred to 20 μl of extraction buffer, consisting of 19 μl of 10 mM Tris, 1 mM EDTA, 1 μl of 0.1% triton X, and 1 μl of proteinase K (20mg/ml, Biolab) into a polymerase chain reaction (PCR) tubes. The nematodes’ cuticle was disrupted by freezing and thawing the sample three times using liquid nitrogen, and then the nematode was incubated overnight at -20 °C. The frozen lysate was incubated at 56 °C for 1 hour followed by 95 °C for 10 minutes for DNA extraction. The PCR reaction was performed with a final volume of 25 μl, which consists of 12.5 μl of EconoTaq PLUS 2X Master Mix (Lucigen, LGC Biosearch Technologies in Madison, WI, USA); 2 μl genomic DNA; 8 µL of nuclease-free water; and 1.25 µL each of forward and reverse primers at a concentration of 10 µM. The primers amplifying the D2-D3 expansion segments of 28S rRNA were done using D2F: 5'-CCTTAGTAACGGCGAGTGAAA-3' (forward) and 536: 5'-CAGCTATCCTGAGGAAAC-3' (reverse) primers (Nguyen et al. 2006). Similarly, ITS rRNA of Previously identified *T. jeffdanielsi* AD isolate and new *T. jeffdanielsi* LA isolates were amplified using TW81 (5’-GTTTCCGTAGGTGAACCTGC-3’) and AB28 (5’-ATATGCTTAAGTTCAGCGGGT-3’) primers (Hominick et al., 1997) . The PCR condition for both PCR was followed as described by (Campos-Herrera et al., 2011) . Purified PCR products were Sanger sequenced from the forward and reverse strands at the UCR Core Instrumentation Facility according to the manufacturer's protocol.

**Molecular and Morphological characterization**

The sequenced D2/D3 fragment of new isolate was compared to the NCBI database using BLAST. The sequence from the new isolate and other closely related nematode species were aligned using Geneious prime (version 2022.0.1) using default parameters of Clustal Omega. The IQ-Tree program selected TIM3+F+G4 as the best-fit model for constructing a phylogenetic tree. The maximum likelihood (ML) method employing ultrafast bootstrap branch support with 1,000 replicates was used for the phylogenetic tree construction. Morphological analysis of the nematode was performed by mounting the specimen on a glass slide with an agar pad as described by Driscoll in 2008 using Nikon-Eclipse E600 (Nikon, Melville, New York).
