# Corrigendum: First report of Tarantula-parasitic nematode *Tarantobelus jeffdanielsi* from Los Angeles, California

**DOI:** 10.17912/micropub.biology.001130

**Published:** 2024-01-18

## Description

For Baniya, A; Ngov, J; Anesko, K; Dillman, AR (2023). First report of Tarantula-parasitic nematode Tarantobelus jeffdanielsi from Los Angeles, California. microPublication Biology. 10.17912/micropub.biology.000825.In the version of the microPublication originally published online, the name of the species Psalmophoeus iriminia was misspelled. The correct spelling is Psalmopoeus iriminia. This mistake has been corrected online.

